# Influence of stocking density on the growth, immune and physiological responses, and cultivation environment of white-leg shrimp (*Litopenaeus vannamei*) in biofloc systems

**DOI:** 10.1038/s41598-024-61328-4

**Published:** 2024-05-15

**Authors:** Mohamed Mohamed Said, Haitham G. Abo-Al-Ela, Yasmine A. El-Barbary, Omaima M. Ahmed, Hagar Sedeek Dighiesh

**Affiliations:** 1 Department of Aquaculture, Faculty of Fish Resources, Suez University, Suez, 43221 Egypt; 2 Genetics and Biotechnology, Department of Aquaculture, Faculty of Fish Resources, Suez University, Suez, 43221 Egypt; 3 Department of Fish Health and Diseases, Faculty of Fish Resources, Suez University, Suez, 43221 Egypt; 4 Department of Fish Processing and Technology, Faculty of Fish Resources, Suez University, Suez, 43221 Egypt

**Keywords:** Antioxidant response, Bacterial count, Biofloc, Growth, *Litopenaeus vannamei*, Stocking density, Ocean sciences, Animal physiology

## Abstract

Biofloc (BF) stands out as a promising system for sustainable shrimp farming. Optimizing various culture conditions, such as stocking density, carbohydrate source, and feeding management, is crucial for the widespread adoption of the BF system. This study compares the growth performance of white-leg shrimp (*Litopenaeus vannamei*) in culture ponds at low density (LD) with 50 organisms/m^2^ and high density (HD) with 200 organisms/m^2^. Post-larvae of white-leg shrimp were stocked for 16 weeks in both LD and HD groups. The LD group exhibited a superior survival rate, growth rate, and feed consumption compared to the HD group. The BF from the LD system recorded a significantly higher protein content (16.63 ± 0.21%) than the HD group (15.21 ± 0.34%). Heterotrophic bacterial counts in water did not significantly differ with stocking density. However, *Vibrio* count in water samples was higher in the HD group (3.59 ± 0.35 log CFU/mL) compared to the LD group (2.45 ± 0.43 log CFU/mL). The whole shrimp body analysis revealed significantly higher protein and lipid content in the LD group. In contrast, the total aerobic bacterial count in shrimp from the HD group was high, with the identification of *Salmonella enterica* ssp. *arizonae*. Additionally, *Vibrio* counts in shrimp samples were significantly higher in the HD group (4.63 ± 0.32 log CFU/g) compared to the LD group (3.57 ± 0.22 log CFU/g). The expression levels of immune-associated genes, including prophenoloxidase, transglutaminase, penaiedin 3, superoxide dismutase, lysozyme, serine proteinase, and the growth-related gene ras-related protein (*rap-2a*), were significantly enhanced in the LD group. Conversely, stress-related gene expression increased significantly in the HD group. Hepatopancreases amylase, lipase, and protease were higher in the LD group, while trypsin activity did not differ significantly. Antioxidant enzyme activity (catalase, glutathione, glutathione peroxidase, and superoxide dismutase) significantly increased in the LD group. The histological structure of hepatopancreas, musculature, and female gonads remained similar in both densities. However, negative effects were observed in the gills' histology of the HD group. These results suggest that increasing stocking density is associated with significantly negative biological, microbial, and physiological effects on white-leg shrimp under the BF system.

## Introduction

Intensive aquaculture with limited water exchange is a favored new technology for overcoming previous aquaculture constraints related to water and land availability^1^. In biofloc technology (BFT) systems, wasted feed, along with feces, is recycled and renewed into microbial protein. This microbial biomass in BFT systems helps optimize water quality by reducing the water content of nitrogenous compounds^2^.

Systems that do not exchange water limit the selection of cultured species to those that can tolerate multiple stressors, such as turbid water, intermediate dissolved oxygen, and suspended solids^3,4^. Microbial flocs are beneficial for shrimp rearing, as shrimp can graze on them, converting them into animal protein. This is supported by improved feed utilization efficiencies, better growth rates, boosted digestive enzyme activities, and enhanced immune system function^5^. The white-leg shrimp (*Litopenaeus vannamei*), due to its compliance with the mentioned conditions and its high profitability, has become the most commonly cultured species in such systems^6^.

Biofloc (BF) is rich in essential contents such as carbohydrates, proteins, lipids, ash, free amino acids, vitamins, and fatty acids required for aquafeed^7–10^. Shrimp in BFT systems can benefit not only from dry compounded feed but also from heterotrophic bacteria or microorganisms in general, contributing to increased weight gain, stimulated digestive enzyme activity, improved feed conversion rate (FCR), and a boosted immune system ^5^. Despite the reported advantages of shrimp farming with BFT, high stocking density may negatively affect aspects such as growth, feed utilization, water quality, microorganisms, and physiological status^11,12^. Consequently, there may be struggles for food, territory, space, and stresses due to crowding^13,14^.

The immune response in aquatic organisms can be influenced by various factors, including the environment, feed availability, and the genetics of the broodstocks^1,15^. The study by Ekasari et al.^16^ has shown that the use of BFT can activate the immune response in shrimp species like the white-leg shrimp, depending on the sources and amounts of carbon applied in the BFT system. Researchers have also found that the existence of BF communities in the culture medium can promote both growth and the expression of immune-related genes in shrimp post-larvae^17^. BFT has many benefits for aquaculture, enhancing the overall health of farmed species, which could lead to greater efficiency and sustainability in the aquaculture industry. In the case of crustaceans, their innate immunity relies on cellular mechanisms^18^.

Researchers have studied how modulating the food composition, specifically the proportion of macronutrients, can impact digestive biochemistry. This information is relevant to the use of additives like beta-glucan in aquaculture, as it could potentially affect digestive processes and nutrient utilization in aquatic organisms^19–21^.

The shrimp hepatopancreas is a highly susceptible organ to the effects of different pollutants in diets and waterborne substances. This makes it a valuable indicator for monitoring the impact of different toxic substances. Like other creatures, the hepatopancreas in shrimp is particularly useful for assessing its health conditions, resembling a major gland that performs the role of both the liver and pancreas. It consists of tubules lined with various types of epithelial cells^22^.

Other studies have explored BF systems in shrimp^23–25^. However, these studies utilized organic carbon sources such as sugarcane bagasse or rice bran, and/or examined different stocking densities. To gain a more nuanced understanding of how stocking density affects shrimp BFT systems, more specific investigations are necessary. This study seeks to compare growth performance, BF quality, water microbial profile, food quality, and physiological status (including digestive enzymes, immune status, antioxidants, and histology) between two stocking densities: 50 and 200 organisms/m^2^.

## Materials and methods

### Shrimp farming system

The experiment was conducted from July to October 2021, spanning 16 weeks, at a commercial shrimp hatchery in Damietta, Egypt. Six cement ponds, each with a total capacity of 36 m^3^, were utilized. Each pond measured 3 m in width, 10 m in length, and 1.2 m in depth. Sand-filtered seawater (32 ± 0.5 ppt) from the Mediterranean Sea, totaling 30 m^3^, was introduced into each pond. To maintain optimal conditions, the experimental units received continuous aeration and were subjected to a 12-h dark/12-h light cycle. Central air pumps (12 distributors/pond, 5.5 horsepower, Turo Vortex Pumps^®^) facilitated aeration. The aeration line, consisting of 2-inch pipes, was divided into units with flow regulators to fine-tune air compression in all ponds. A network of disk diffusers (each measuring 26 cm) was securely fixed and installed at the bottom of each pond to ensure effective aeration and thorough mixing of the culture water across all ponds^4^.

### Experimental groups and biofloc setup

Post-larvae of white-leg shrimp (*L*. *vannamei*) with a mean body weight of 0.02 ± 0.001 g were stocked at densities of 50 and 200 organisms/m^2^, with triplicates for each treatment. To establish the BF, we followed the method outlined by Said et al.^4^. Wheat flour, containing 58.3% carbon, was introduced once daily to all experiments as a carbohydrate source to stimulate the development of the BF community^26^. Wheat flour was chosen due to its efficient utilization and positive impact on water quality parameters when used in BF systems ^27,28^. The carbon-to-nitrogen (C:N) ratio was maintained at 15:1 by adding the necessary amount of the carbon source. The pre-weighed carbon supplement was mixed with pond water and evenly distributed across the surfaces of the ponds.

### Management of experimental units

Post-larvae were fed four times a day at 7:00 AM, 11:00 AM, 2:00 PM, and 5:00 PM with a commercial shrimp feed (Skretting^®^, El-Sharkia, Egypt) containing 38% crude protein. The proximate analyses of both the feed and carbon bases used in this study are detailed in Table 1. The post-larvae were fed daily as a percentage of the total biomass, with daily feeding rates gradually decreasing from 32 to 1.9% over the experimental period.

**Table 1:** Proximate analysis of the experimental feed and carbon sources (wheat flour).

Constituent	Feed	Wheat flour
Crude protein (CP%)	38.86 ± 1.03	10.93 ± 0.16
Ether extract (EE%)	8.99 ± 0.13	3.03 ± 0.04
Crude fiber (CF%)	4.39 ± 0.09	5.33 ± 0.15
Total Ash (%)	8.99 ± 0.11	1.58 ± 0.03
Moisture (%)	8.55 ± 0.09	10.90 ± 0.12
Nitrogen free extract (NFE%)	30.22 ± 1.09	68.23 ± 0.08

Values are presented as means ± SE. NFE = % DM—(CP + EE + CF + ASH). Data are expressed as means ± SE.

Feed quantities were adjusted every two weeks based on Van Wyk^29^, taking into account any observed mortality. Monitoring of temperature, dissolved oxygen, nitrite, ammonia, BF volume, and turbidity was conducted daily throughout the experiment. The HANNA^®^ HI9146-04 electronic probe was employed for measuring water dissolved oxygen and temperature. Ammonia and nitrite levels were assessed using the HANNA^®^ HI97715 photometer, while the Milwaukee^®^ MW102 portable pH meter was utilized for measuring water pH. Turbidity was monitored with a turbidity meter from Lovibond^®^ (TB211 IR), and floc volume was determined using an Imhoff cone.

### Assessment of growth performance and survival rate

Upon completion of the experiment, shrimp was collected after pond draining. The shrimp count was recorded to assess survivability in each pond. Specific growth rate (SGR; %), final individual weight (FW; g), weight gain (WG; g), weight gain per week, total biomass, and biomass weight gain % were calculated to evaluate the growth performance. Feed exploitation was characterized by calculating the feed conversion ratio (FCR) and protein efficiency ratio (PER). Growth, survival, and feed utilization were calculated based on Tacon et al.^30^.

### Macronutrient analysis of shrimp and biofloc

The composition of BF communities and shrimp whole bodies was assessed following the guidelines of the Association of Official Analytical Chemists^31^. At the conclusion of the experiment, shrimp samples were collected from each pond. BF masses were collected using a 100 mm mesh net for subsequent biochemical analysis. These samples were then dehydrated in a Nabertherm^®^ heating oven at 60 °C, ground, and subjected to biochemical analysis.

Moisture content was determined by drying a fixed quantity of samples (5 g) at 105 °C in a binder heated oven until a consistent weight was achieved. Ash content was estimated by burning a fixed number of dried samples in a muffle furnace at 550 °C for 4 h, followed by cooling and weighing.

Crude proteins were quantified using the Kjeldahl method (FOSS^®^, KjelTec^™^ 84,000). Fat extraction was performed using an automated fat extraction system (FOSS^®^, Soxtec^™^ 8000). The estimation of crude fiber employed an automatic fiber analyzer system (FOSS^®^, Fibertec^™^ 8000), while the nitrogen-free extract was calculated by taking the difference.

### Bacterial analysis

At the conclusion of the experiment, ten randomly selected shrimp and 100 mL water samples were collected from each pond. These samples were transported aseptically to the fish microbiology laboratory at the Fish Resources Faculty, Suez University, and were examined immediately.

Shrimp samples were prepared, and heterotrophic and *Vibrio* bacterial counts were estimated following the FDA guidelines^32^. Colonies of the selected bacteria underwent Gram staining and were identified biochemically using indole, Voges–Proskauer, and methyl red tests. Suspected *Vibrio* colonies were subjected to oxidase biochemical tests and API 20E diagnostic strips^33,34^. Bacterial species were then confirmed using API 20E strips purchased from BioMérieux, France^35,36^.

### Expression analysis of immune-, stress-, and growth-related genes

At the conclusion of the experiment, three organisms from each group underwent hemolymph sampling for total RNA extraction using TRIzol reagent (QIAzol^®^, QIAGEN). Synthesis of cDNA from 1.5 μg of RNA was accomplished using the Revert-Aid First Strand cDNA Synthesis Kit^®^ (Thermo Fisher Scientific).

Specific primers (Table 2) were employed for conducting real-time PCR of immune- and stress-associated genes, including prophenoloxidase (*propo*), transglutaminase II (*TGaseII*), superoxide dismutase (*sod*), lysozyme (*lyz*), penaiedin 3 (*pen 3*), serine proteinase (*sp*), and heat shock protein 70 (*hsp70*). The growth-associated gene was represented by ras-related protein (*rap-2a*). Real-time PCR was performed using SYBR Green master mix (Top Real SYBR mix^®^, Biovision). The PCR conditions were as follows: a denaturation step at 95 °C for 15 min, followed by 40 cycles at 95 °C for 15 s, then a 60 °C/30 s step, and finally 72 °C for 30 s.

**Table 2:** Sequences of primers used in real-time PCR.

Gene	(5′–3′) primer sequence	NCBI reference/accession number
Prophenoloxidase (*propo*)	F: GAGATCGCAAGGGAGAACTG R: CGTCAGTGAAGTCGAGACCA	EF565469.1
Serine proteinase (*sp*)	F: CGTCGTTAGGTTAAGTGCGTTCT R: TTTCAGCGCATTAAGACGTGTT	AY368151.1
Transglutaminase (*TGase II*)	F: CCTCAGGATCTCCTTCACCA R: TTGGGAAAACCTTCATTTCG	EU164849.1
Lysozyme (*lyz*)	F: GAAGCGACTACGGCAAGAAC R: AACCGTGAGACCAGCACTCT	AF425673.1
Superoxide dismutase (*sod*)	F: ATCCACCACACAAAGCATCA R: AGCTCTCGTCAATGGCTTGT	DQ298207.1
Penaiedin 3 (*pen 3*)	F: GCCCGTTACCCAAACCATC R: CCGTATCTGAAGCAGCAAAGTC	DQ211701
Heat shock protein 70 (*hsp70*)	F: GGCAAGGAGCTGAACAAGTC R: TCTCGATACCCAGGGACAAG	JQ736788
Ras-related protein (*rap-2a*)	F: GCCGTGCGTGCTTGAGAT R: TTGATGTCCTGGAAGGTCTGG	XM_027358136.1
*β*-actin	F: CCACGAGACCACCTACAAC R: AGCGAGGGCAGTGATTTC	AF300705

Gene expression was calculated using the *β*-actin gene as an internal reference. Expression results were presented as fold change using the 2^−ΔΔCT^ method according to Livak and Schmittgen^37^ replacing control values with higher density values. Data are present as fold changes compared to higher density (200 organisms/m^2^).

### Digestive enzymes

The pooled hepatopancreases of shrimp were weighed, homogenized with sterilized distilled water, balanced, and then uniformly mixed with chilled phosphate buffer (0.65%, 1:10 w/v, pH 7). The resulting supernatants from centrifugation (800 × *g* for 10 min at 4 °C) were utilized for various digestive enzyme assays. Supernatants without a lipid coat were separated for each specific enzyme activity test. The homogenized dilutions were completed with buffers and tested in duplicate. Digestive enzyme measurements were expressed as enzyme units per gram of tissue.

Amylase activity was assessed following the procedure of Rick and Stegbauer^38^, known as the ‘3, 5-dinitrosalicylic acid’ method. Amylase activity was determined from the maltose standard curve, with the amount of maltose obtained from starch/min/mg protein at 37 °C. Protease activity was measured according to Drapeau^39^ using the casein digestion method. Tyrosine release was estimated from the standard curve, indicating one micromole of tyrosine as unit/mg protein at 37 °C. Lipase enzyme activity was determined by the milliequivalents of alkali consumed. Trypsin enzyme activity was assessed following the technique of Zhang et al.^40^ with casein substrate. One unit of trypsin was defined as the number of micromoles of tyrosine freed per minute per milligram of protein.

### Antioxidant enzymes

About 200 μL of pooled hemolymph was withdrawn from the ventral sinus of a shrimp, located at the bottom of the primary abdominal segment. This hemolymph was then transferred into a 1 mL sterilized syringe and mixed with an anticoagulant mixture. The mixture comprised 0.34 M sodium chloride, 30 mM trisodium citrate, and 10 mM EDTA-Na_2_, as detailed by Xu and Pan^41^. The resulting mix was adjusted to a pH of 7.55 and an osmolality of 780 mOsm kg^−1^.

Next, the anticoagulant-hemolymph mix from 10 shrimp in each pond was pooled, gently mixed, and subjected to centrifugation at 800 × *g* for 10 min at 4 °C. The supernatant obtained after centrifugation was withdrawn as plasma samples and stored at − 80 °C for subsequent antioxidant analysis.

For the assessment of superoxide dismutase (SOD) activity, the “hydroxylamine method” outlined by Xu and Pan^42^ was employed. Catalase activity was determined using the “visible light method” as described by Kim et al.^43^, while the “turbidimetric method” specified by Feng et al.^44^ was utilized for measuring glutathione peroxidase activity.

### Histological examination

Thirty samples, comprising 15 from each group, were collected. The gills, intestine, stomach, hepatopancreas, and female gonad were promptly excised and fixed in a 10% formalin solution for 24 h. Subsequently, the samples underwent a thorough process, including washing with distilled water, dehydration with a series of ethyl alcohol concentrations (70%, 80%, 90%, 95%, and 100%), overnight immersion in methyl benzoate, and two rounds of clearing with xylene. Following these preparations, the samples were fixed in 65–70 °C paraffin, sliced into sections of 5–6 μm, and stained with hematoxylin/eosin. The examination of the samples was conducted using a Leica^®^ ICC50 HD light microscope.

### Statistical analysis

The impact of various stocking densities on different parameters was analyzed using the t-test in IBM SPSS Statistics version 26 (IBM Corporation, NY, USA). Results were presented as mean ± SE, and differences in means were assessed using Duncan’s multiple-range test. A probability value of < 0.05 was employed to signify statistically significant differences.

### Ethical approval

The study has been approved by the Faculty of Fish Resources, Suez University, under the protocol of international and national guidelines for the ethical treatment of animals.

## Results and discussion

### Water quality, growth performance, survival rate, and feed utilization

All measured water quality parameters, including dissolved oxygen (DO), pH, NH_3_, NO_2_, and turbidity, did not exceed the appropriate levels for white-leg shrimp culture^45^ in both groups throughout the entire experimental period (Table 3).

**Table 3:** Water quality parameters.

Parameter	Mean ± SE
Temperature (°C)	27.1 ± 2.2
DO (mg/L)	5.59 ± 0.06
NH_3_ (mg/L)	0.03 ± 0.00
NO_2_ (mg/L)	0.33 ± 0.01
Ph	7.09 ± 0.07
Turbidity (NTU)	55.72 ± 2.23
Salinity (PPT)	32 ± 0.78
Floc volume (mL/L)	17.19 ± 0.58

DO (dissolved oxygen); NH_3_ (ammonia); NO_2_ (nitrogen dioxide). Data are expressed as means ± SE.

Significant differences in growth-performance parameters and survival rates were observed between the two densities (Table 4). The final individual weight increased by 5.66 g in the lower density (LD) compared to the higher density (HD). Similarly, specific growth rates (SGR%) showed a significant increase of 0.32%/day in the LD group. Weekly growth was significantly enhanced in the LD group, with a biomass weight gain percentage showing a significant increase of 292.72 compared to HD. Notably, the survival rate in the LD group was approximately 99%, compared to 97% in the HD group. In contrast, total biomass was higher in the HD group than in the LD group (Table 4).

**Table 4:** Growth performance, survival rates, and feed utilization of white-leg shrimp (*Litopenaeus vannamei*) over a 16-week period, with variations in stocking densities within a biofloc system.

Parameter	Stocking density		*p* value
	50 organisms/m^2^	200 organisms/m^2^	
Final weight (g)	18.5 ± 0.06	12.84 ± 0.06	< 0.01
Weight gain (g)	18.48 ± 0.06	12.82 ± 0.06	< 0.01
Specific Growth rate (g)	6.09 ± 0.00	5.77 ± 0.00	< 0.01
Growth/week (g)	1.15 ± 0.00	0.80 ± 0.00	< 0.01
Biomass (kg)	27.42 ± 0.18	74.53 ± 0.56	< 0.01
Biomass gain (%)	913.66 ± 5.87	620.94 ± 4.46	< 0.01
Survival rate (%)	98.78 ± 0.11	96.75 ± 0.29	< 0.01
Feed conversion rate	1.20 ± 0.01	1.36 ± 0.00	< 0.01
Protein efficiency ratio	2.17 ± 0.03	1.93 ± 0.01	< 0.001

Data are expressed as means ± SE.

Acceptable growth performance and high survival rates were achieved in both systems, even at higher density, in agreement with El-Sayed^46^. The BF system demonstrated greater holding capacity than conventional clear water aquaculture^28^. The high survivability and growth in the BFT systems may be attributed to favorable environmental conditions, coupled with the high nutritional value of flocs for shrimp. Additionally, microbial communities within the flocs could enhance the activity of digestive enzymes, gastrointestinal microflora, and overall nutrient utilization^41^. Successful super-intensive culture in BFT systems for rearing white-leg shrimp at HD reflects the species' ability to achieve better growth performance and higher survival^47^.

The current results revealed that the higher stocking density treatment led to lower growth performance and survival compared to the lower density. A negative relationship between stocking density and shrimp growth had been previously documented^11,48–50^. This could be attributed to the energy expended on food competition and the stress caused by a larger number of animals^13,51^. Fleckenstein et al.^47^ reported that higher final weight and faster growth rates were achieved in LD treatments (100 shrimp/m^3^) compared with 200 shrimp/m^3^. Additionally, LD showed slightly higher survival (94.5%) compared to HD (91.3%).

The results of the current study showed a significantly higher total biomass in the higher stocking density treatment, aligning with Fleckenstein et al.^47^. These authors reported significantly superior total shrimp biomass production in the HD group (4.0 kg/m^3^) compared to the LD group (2.3 kg/m^3^). The notable increase in biomass production in high-density stocking rates could provide a solid rationale for using elevated shrimp densities.

In the current study, improved feed efficiency (FE) was observed in the LD group (50 shrimp/m^2^) compared to the HD (200 organisms/m^2^). Additionally, the protein efficiency ratio (PER) increased by 0.24 in the LD group compared to the HD group (Table 4). Fleckenstein et al.^47^ reported low FCR values, ranging from 1.0 to 1.1 in low-density (2.3 kg/m^3^) BF treatments. The enhanced feed utilization in BF systems may be attributed to indications that the presence of BF communities could elevate enzymatic activity in the digestive canal. Consequently, this increase in enzymatic activity is contributing to improved feed efficiency and reduced FCRs^52^.

Vungarala et al.^12^ and Nguyen et al.^53^noted higher FCR values with increased stocking densities of white-leg shrimp. The observed better feed utilization parameters in the lower density system indicate a reduced ability of shrimp to graze on the flocs at high density^24^. The higher PER value in the LD system could be explained by the sustained availability of protein-rich BF particles, which can be consumed by the shrimp, and/or the stressful conditions in the high-density system (crowding, less favorable water quality, etc.)^54,55^.

### Analysis of macronutrients in biofloc and shrimps

A proximate analysis of BF composition taken from the LD group revealed a numerical superiority in lipid content (Table 5). Additionally, significantly higher protein and carbohydrate contents were noted in the LD group, while ash and fiber contents from the HD system (200 organisms/m^2^) were both higher than those in the LD system (50 organisms/m^2^) (Table 5).

**Table 5:** Proximate analysis of white-leg shrimp (*Litopenaeus vannamei*) and biofloc particles collected from biofloc systems with varying stocking densities.

Biofloc particles	Stocking density		*p* value
	50 organisms/m^2^	200 organisms/m^2^	
Fat (%)	1.65 ± 0.26	0.93 ± 0.06	0.10
Protein (%)	16.63 ± 0.21	15.21 ± 0.34	0.02
Fiber (%)	13.8 ± 0.17	17.1 ± 0.32	< 0.01
Ash (%)	15.06 ± 1.20	19.63 ± 0.54	0.02
Carbohydrate (%)	52.84 ± 0.86	47.12 ± 0.52	< 0.01

Shrimp	Stocking density		*p* value
	50 organisms/m^2^	200 organisms/m^2^	
Fat (%)	4.36 ± 0.08	3.00 ± 0.20	< 0.01
Protein (%)	73.9 ± 0.34	71.76 ± 0.37	0.01
Ash (%)	12.46 ± 0.08	14.56 ± 0.57	0.02
Carbohydrate (%)	0.56 ± 0.31	1.64 ± 0.88	0.31

Data are expressed as means ± SE.

BF particles possess good nutritional value and can be used as aquatic food along with artificial feed^56^. The results of the present study demonstrated an enhanced nutritive value of the BF in the LD system. Various proteins, lipids, and ash contents in the BF particles were found in previous studies. Tao et al.^57^ found that the average protein content in floc particles gathered at different stocking densities varied from 28.9 to 29.2%. Khoa et al.^58^ recorded that protein levels in floc particles ranged from 22.2 to 25%. Regarding lipid content, Tao et al.^57^ found that lipid contents in floc particles ranged from 5.4 to 5.7%. Additionally, different ash content in flocs was reported; for example, Tao et al.^57^ found that ash contents were approximately 29.1% without differences in all stocking densities, and these differences might be due to the diversity in carbon sources applied^59^.

The nutritional value of BF may vary between treatments due to changes in the microbial community. Floc size can also influence its nutritional composition; Ekasari et al.^10^ and Ray et al.^60^ reported increased protein and lipid content in floc with an increase in floc particle size. Additionally, C:N ratios can modify the microbial and nutritional composition of BF. Panigrahi et al.^61^ reported that the C:N ratios (5; 10; 15; 20) had a significant impact on the developmental growth and characteristics of BF.

The whole-body proximate composition of shrimp showed significantly higher protein and lipid content in the LD compared to the HD treatment (Table 5). Similar to the BF analysis, significant ash content was found in shrimps of the HD system. However, the two treatments were not significantly different in fiber and ash contents (Table 5).

High contents of crude proteins, lipids, and ash were observed in the shrimp cultured in the BFT. Rajkumar et al.^28^ recorded that using carbohydrates in the BFT caused a surge in protein utilization efficiency and supported essential lipids and vitamins for the shrimp. The change in the body composition of the shrimp may be attributed to various factors such as water quality, stress, nutrient abundance, and feed intake and utilization^62^. The decreased lipid content in the HD system compared to the LD system can be a result of reduced feed intake and low amounts of accumulated lipids in the carcass structure^63^. However, another study reported that different densities had no significant differences in the high content of crude protein, lipid, and ash in the shrimp in the BFT^12^. Also, no variations in nutritional contents were observed in Nile tilapia cultured in a BF using diverse stocking densities^64^.

### Bacterial analysis

Heterotrophic bacterial counts were higher in the LD water than those detected in the HD water (*p* > 0.05; Table 6). The elevated bacterial counts in water samples at both densities were expected, as the BFT system enhances the multiplication of advantageous heterotrophic bacteria^65–67^. Similar results were reported by Arias-Moscoso et al.^68^, where heterotrophic bacterial counts in the shrimp-rearing BF system at day 30 ranged from 38.2 to 65.3 × 10^6^ CFU/mL.

**Table 6:** Heterotrophic, *Vibrio*-like bacterial count, and other bacteria isolated from water (log CFU/mL) and white-leg shrimp (*Litopenaeus vannamei*) samples (log CFU/g) in biofloc systems with varying density.

	Water			Shrimp		
	50 organisms/m^2^	200 organisms/m^2^	*p* value	50 organisms/m^2^	200 organisms/m^2^	*p* value
Heterotrophic bacterial count	5.10 ± 0.28	5.70 ± 0.09	0.08	4.85 ± 0.14	5.77 ± 0.15	0.03
*Vibrio*-like bacterial count	2.45 ± 0.43	3.59 ± 0.35	0.02	3.57 ± 0.22	4.63 ± 0.32	< 0.01

Data are expressed as means ± SE.

In contrast, the *Vibrio* bacterial count in the water was significantly higher in the HD than in the LD (*p* = 0.02; Table 6). Arias-Moscoso et al.^68^ also reported similar findings on day 30, ranging from 1.67 to 4.23 × 10^3^ CFU/mL, through the supplementation of commercial probiotics in a shrimp BF system. *Enterobacter cloacae* and *Enterobacter amnigenus* were identified in this study in the water of both HD and LD systems. *Enterobacter* spp. is a natural commensal of the animal gastrointestinal tract microorganisms^69^. Its presence in water samples in both studied system densities might be attributed to the accumulation of shrimp waste in the BF system.

Shrimp samples were analyzed to assess the effect of variable densities on shrimp food bacterial quality. The total bacterial counts in the HD system documented about a 1-log increase compared to the LD system. Similarly, the *Vibrio*-like bacterial count was markedly higher in shrimp samples from the HD compared to the LD (Table 6). The same patterns of *Vibrio*-like bacterial counts were found in the water from both systems, which might reflect on shrimp. *Vibrio* bacteria naturally exist in water environments and the gastrointestinal tract of shrimp^70^. Additionally, pathogenic *Vibrio* spp. causing food-borne infections were not identified in water or shrimp in both systems, while pathogenic *Salmonella enterica* subsp. *arizona* was identified in shrimp samples from the HD system (Table 6).

### Gene expression

**Figure 1:**
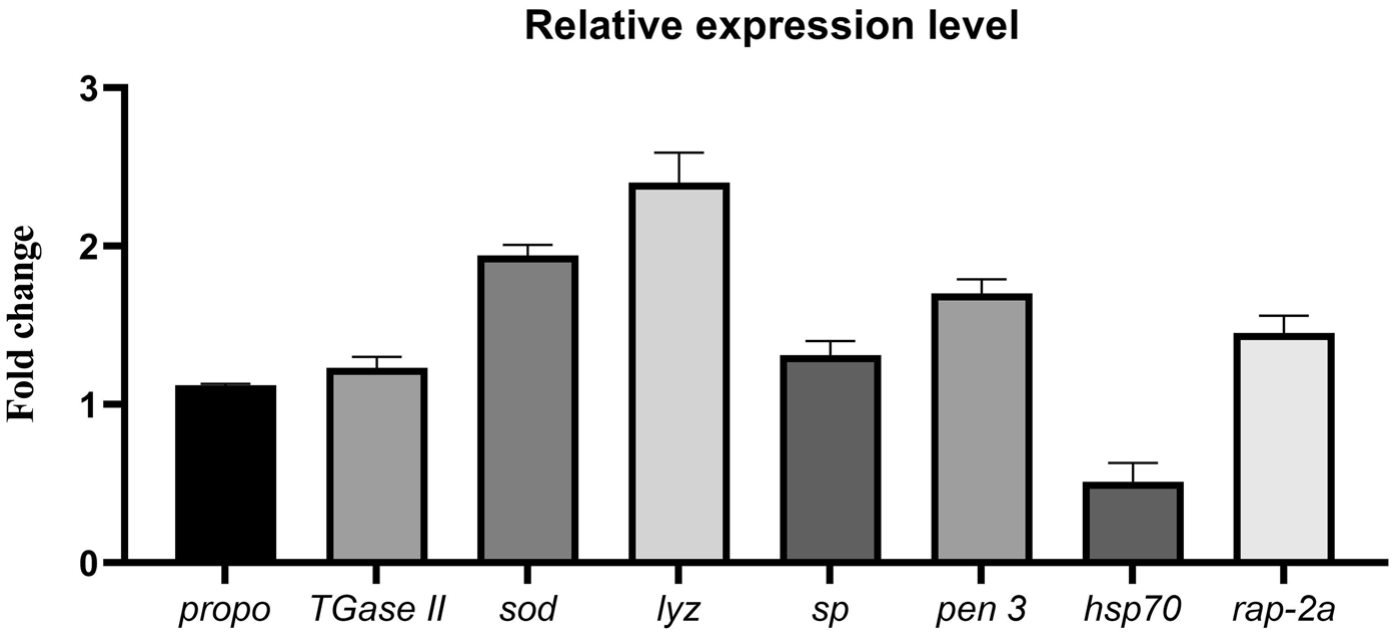
Fold change in expression levels of prophenoloxidase (*propo*), transglutaminase II (*TGaseII*), superoxide dismutase (*sod*), lysozyme (*lyz*), serine proteinase (*sp*), penaiedin 3 (*pen 3*), heat shock protein 70 (*hsp70*), and ras-related protein (*rap-2a*) in the hemolymph of white-leg shrimp (*Litopenaeus vannamei*) under two different rearing conditions: low density (50 organisms/m^2^) in a biofloc system and higher density (200 organisms/m^2^). A fold change less than 1 indicates downregulation, while a fold change greater than 1 indicates upregulation compared to the higher density condition (200 organisms/m^2^). Data are presented as means ± SE.

Generally, BFT contains a plentiful count of bacteria, and their cell wall consists of lipopolysaccharide, β-1, 3-glucans, and peptidoglycan. Consequently, it exhibits a probiotic effect and is recognized as a stimulant for the non-specific immunity of shrimps^17,54^. The expression levels of immune-related genes, namely *propo*, *TGase II*, *sod*, *lyz*, *sp*, *pen 3*, and the growth-related gene *rap-2a* in shrimps of the LD group were significantly higher than those of the HD group (Fig. 1). Conversely, the expression of the stress-related gene *hsp70* showed a significant increase in the HD group (Fig. 1).

The proPO-system is activated upon the recognition of specific structures of probiotics or pathogens by pattern recognition proteins. It involves a serine proteinase pathway that ultimately cleaves and activates the proPO^71^. The respiratory burst of hemocytes aids in the clearance of shrimp infections and is linked to the release of numerous reactive oxygen intermediates (ROIs)^72–74^. ROIs are considered essential for host defense, but their overexpression could potentially harm host cells^75^. SOD functions to maintain the lowest practical levels of ROIs intracellularly^76^. The documented impact of probiotics and BFT on enhancing the shrimp's respiratory burst has been reported^10,77,78^.

TGase is known to play a crucial role in the mechanism of blood coagulation, implicated in the defense mechanisms of invertebrates. TGase is broadly expressed in hematopoietic tissue and hemocytes, and the presence of lipopolysaccharide induces the rapid release of TGase as a response of hemocytes^79,80^. The upregulation of TGase in the LD group, along with the elevated *lyz* expression level, suggests that TGase is a significant component in the activation of the shrimp’s immune response. Additionally, it is implicated in the modulation of antimicrobial peptides such as lysozyme^79^.

Other groups of antimicrobial peptides, including the penaeidins, are primarily expressed in hemocytes and provide defense against pathogens and infections^81,82^. Moreover, they contribute to opsonization by labeling bacterial surfaces, thereby enhancing immune reactivity and facilitating the elimination of marked antigens through phagocytosis^83^. Similarly, the expression levels of entire antimicrobial peptide gene groups were upregulated when bacterial probiotics were administered to shrimp, as documented by Antony et al.^84^.

In the higher-density group, *hsp70* expression was significantly upregulated, indicating a correlation between shrimp stocking density and stress. This correlation is supported by Liu et al.^54^, who highlight the link between stocking density and stress. The evidence suggests that a *Bacillus* species mix can reduce cellular stresses in seabream larvae by decreasing the expression of *hsp70*, enhancing the fish's tolerance toward culture conditions^85^. Feed additives, immuno-stimulants, prebiotics, and probiotics are widely used in aquatic organisms to alleviate stress and enhance immune status^86,87^.

### Digestive enzymes

**Figure 2:**
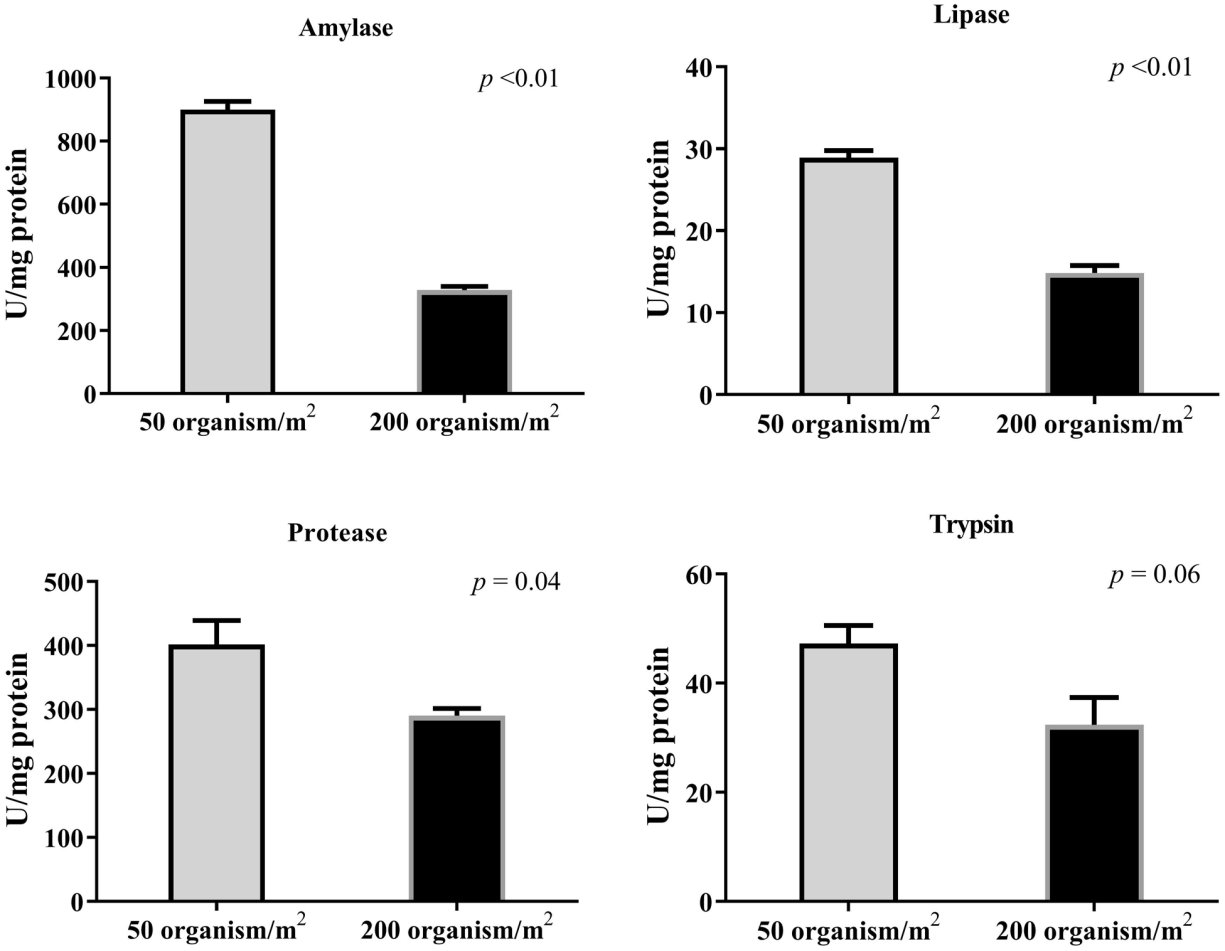
Activity of hepatopancreas digestive enzymes (amylase, lipase, protease, and trypsin) of white-leg shrimp (*Litopenaeus vannamei*) reared under varying stocking densities using biofloc system. Data are expressed as means ± SE.

Amylase, lipase, protease, and trypsin activities were estimated in the shrimp's hepatopancreases at the end of the experimental period (Fig. 2). The activities of enzymes (amylase, lipase, and protease) were significantly higher in the LD group than in the HD group, while no significant differences were observed in the activity of trypsin.

Various factors may influence the effectiveness of digestive enzymes in both fish and shrimp. Shao et al.^88^ reported significant differences in trypsin activity in white-leg shrimp hepatopancreas samples fed different diets, with no notable differences in amylase and lipase activities. For instance, shrimp and fish fed with probiotics, microalgae, and periphyton-supplemented diets experienced increased levels of digestive enzyme activity^89,90^. An advantage of the BF system is its ability to enhance the digestibility capacity of reared shrimp by boosting the activity of digestive enzymes. The activities of digestive enzymes are critical factors for optimizing nutritional procedures and improving digestibility in aquatic animals, facilitating the breakdown and absorption of nutrients, growth enhancement, and adaptability to the environment^91,92^. This is consistent with the current results; the activities of the digestive enzymes amylase and lipase were lower in the HD (500 shrimp/m^3^) group than in the LD (300 shrimp/m^3^) group^54^.

### Antioxidant enzymes

**Figure 3:**
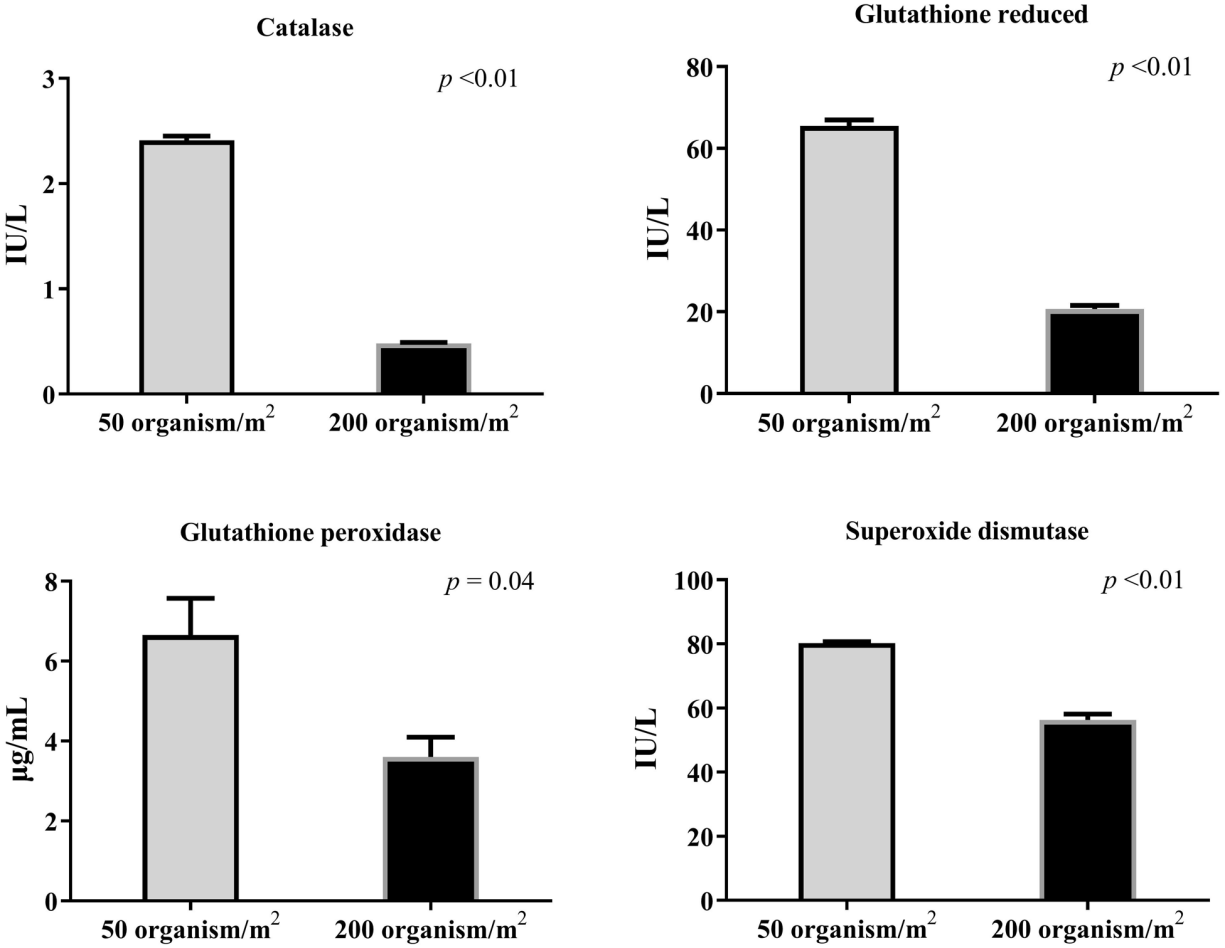
Activity of antioxidant enzymes (catalase, glutathione reduced, glutathione peroxidase, and superoxide dismutase) in the hemolymph of white-leg shrimp (*Litopenaeus vannamei*) reared under varying stocking densities using biofloc system. Data are expressed as means ± SE.

Antioxidant enzymes, including SOD and glutathione peroxidase, constitute the primary enzymatic defense against free radicals in organisms. When their levels decline, free radicals increase, leading to impairment of cell function^93^. In this study, antioxidant enzymes (catalase, SOD, glutathione reduced, and glutathione peroxidase) significantly increased in shrimp reared under the LD compared to the HD group (Fig. 3).

Conditions in the BF system positively contribute to the antioxidant activity in shrimp. Xu and Pan^5^ observed that the antioxidant activity in shrimp subjected to two BF treatments was superior to that in the control group. Furthermore, Liu et al.^54^ concluded that immunological response parameters and antioxidants, including SOD, glutathione peroxidase, and malondialdehyde, all decreased in higher density conditions (300 and 400 shrimp/m^2^). Emerenciano et al.^9^ noted that BFT with a lower density system improved the antioxidant state of shrimp and reduced oxidative stresses, leading to better stress management during transfer into the hatchery and the reproduction process. The lower stocking density group exhibited lower oxidative stress levels compared to the higher stocking density group. These findings align with gene expression results, indicating a robust immunological and antioxidant response in low-density stocking.

### Histological assessment

**Figure 4:**
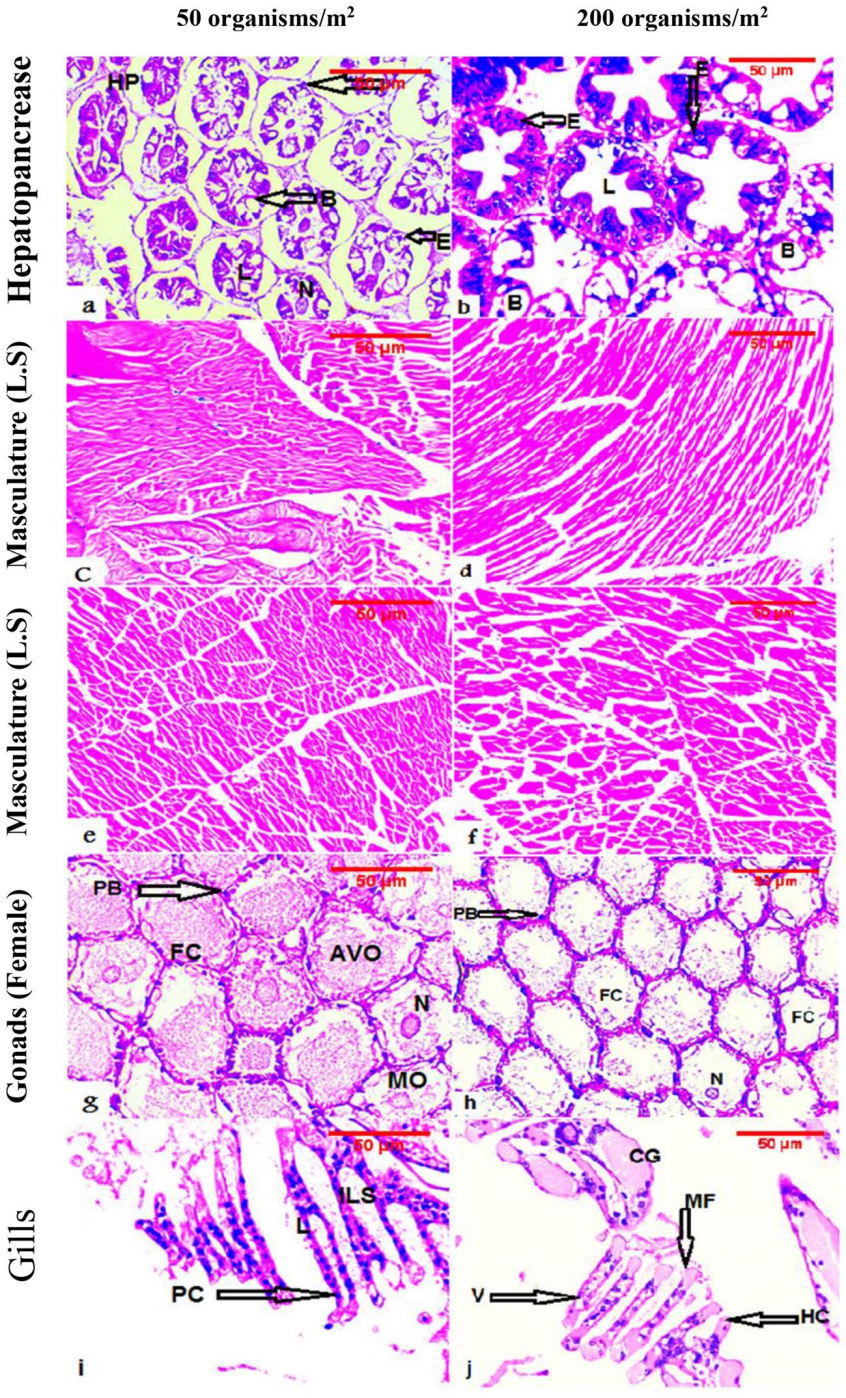
Hepatopancreas, musculature (longitudinal (L.S.) and transverse sections (T.S.)), female gonad, and gills of white-leg shrimp (*Litopenaeus vannamei*) that were cultured under varying densities through a biofloc system for a duration of 16 weeks.

Histological assessment (Fig. 4) revealed positive effects of the BF system on the health and structure of organs, as observed in the hepatopancreas, musculature, and gonad. Conversely, histological examination of gills indicated negative effects in the HD group, impacting the gill structure (Fig. 4).

The hepatopancreas histology exhibited healthy hepatopancreas cells, lumen, nucleus, connective tissue, Blasenzellen cells (B), and Embryonal-Zellen cells. Musculature longitudinal and transverse sections displayed a normal structure. The female gonad showed a developed stage with peripheral cells, follicular cells, advanced vitellogenic oocyte, nucleus, and mature oocyte. In contrast, the gills in the lower density group maintained a normal structure with lamellae, uniform interlamellar space, and pillar cells. The higher density group, on the other hand, exhibited abnormal gill structure and lamellae with vacuoles, accumulation of hemocytes, abnormal gill tips, malformation of gill tips, and clubbing lamellae.

The developmental status of the hepatopancreas can serve as a vital indicator of its functional activity^94^. The hepatopancreas performs essential functions such as lipid storage, nutrient absorption, and digestive enzyme production^95^. Blasenzellen (B) cells within the hepatopancreas are the primary and largest producers of digestive enzymes, responsible for nutrient accumulation, intracellular digestion, and transportation of digested material^96^. Shi et al.^97^ demonstrated that the dietary microbial-derived antioxidant can enhance hepatopancreas functional activity through improved enzyme production. BF, recognized as a high-quality continuous food source, has been associated with improved production outcomes, shorter growth times, better survival rates, and well-structured musculature^98,99^.

The energy required for reproduction may be higher in females reared under BFT, and reduced glutathione levels are critical for organisms facing occasional oxidative stress^97,100^. Xu and Pan^41^ highlighted the beneficial effects of BF as a potential dietary source of antioxidants for white-leg shrimp. It is evident that BFT enhances reproduction, aligning with the histological results of female gonad development in both treatments.

Shrimp gills play a crucial role in breathing, osmotic regulation, and ionic regulation^101^. Obstruction of gills by microorganisms as well as accumulation of inorganic nitrogen compounds can adversely affect shrimp breathing and osmoregulation by damaging the organ's structure^102,103^. Abnormal gill structure, such as malformation and clubbing lamellae, was evident in the higher-density group. In intensive aquaculture systems with no water exchange, shrimp mortality was linked to gill occlusion and high solid content in the water^103,104^. Total suspended solids and BF levels were found to positively correlate with stocking density, leading to an increased incidence of shrimp with stuffed gills^104^. Fregoso-López et al.^103^ associated histological changes with more damage in shrimp gills at elevated stocking density, contributing to shrimp mortality. These findings may explain the significantly advanced survival rate in the lower-density group in the current study.

## Conclusion

The impact of stocking density on shrimp farming using a BF system was significant. A comparison between two BF systems, one with a stocking density of 50 shrimp/m^2^ and the other with 200 shrimp/m^2^, highlighted several drawbacks associated with higher stocking density. Growth performance parameters were reduced, the FCR increased, and the survival rate decreased. Additionally, higher counts of *Vibrio*-like bacteria in water and shrimp, along with the detection of *Salmonella enterica* ssp *arizonae*, were observed. A decline in body composition was also noted. Moreover, immune and growth-related genes showed lower expression, while stress genes exhibited increased expression. Reduced activity of digestive enzymes, antioxidants, and abnormal histological gill structure were additional findings. Further studies exploring varied inputs and operational methodologies for high-density BF systems could lead to highly productive production without undesirable consequences.

## Data Availability

The datasets produced and analyzed in this study can be obtained from the corresponding author upon a reasonable request.
